# Early-stage chronic venous disorder as a cause of leg pain overlooked for lumbar spinal disease

**DOI:** 10.1038/s41598-023-45623-0

**Published:** 2023-10-25

**Authors:** Dong-Hoon Yang, Mansu Kim, Joong Won Yang, Jae Man Cho, Sang Jin Park, Heum Dai Kwon

**Affiliations:** Department of Neurosurgery, Pohang Stroke & Spine Hospital, 352 Huimang-daero, Namgu, Pohang, Gyeong-sang bukdo 37659 Republic of Korea

**Keywords:** Chronic pain, Peripheral vascular disease

## Abstract

Leg pain can be caused by both lumbar spinal disease and chronic venous disorder (CVD) of leg veins, but their clinical differences have not been thoroughly investigated. This study aimed to determine the incidence of CVD among patients visiting a spine center for leg pain. A total of 196 cases underwent ultrasound examination with a diagnosis rate were 85.7% (168 cases). CVD-diagnosed cases were divided into two groups based on the severity of lumbar spinal disease. The Clinical grades, symptom areas, and symptom types were compared. The differences in symptom improvements with vasoactive medication were also assessed. The most common symptom area was calf then the foot in CVD, while calf then thigh in lumbar spinal disease. Tingling-paresthesia was the most common symptom type for both, with pain and cramping similarly common in CVD and pain more common than cramping in lumbar spinal disease. Considering that the majority of CVD cases (78.6%) had minor cutaneous changes and almost half of cases (41.7%) had refluxes only in tributaries, significant differences in symptom improvement in CVD-dominant group suggested that early-stage venous reflux is a symptomatic disease and a possible cause of leg pain and other symptoms.

## Introduction

Lumbar disc herniations and other degenerative diseases are common causes of leg pain, tingling, sensory change, and weakness. Patients with leg pain usually seek relief from lumbar spinal pain by visiting spine centers. However, not every patient is fortunate enough to receive a clear lumbar diagnosis. Leg pain can be caused by other disease conditions such as arterial occlusion, peripheral neuropathy, deep vein thrombosis, knee or hip arthrosis, cellulitis, Baker’s cyst, muscular injury, tumor or infection, arterial aneurysm, and Achilles tendon inflammation or rupture^1–4^.

CVD is caused by chronic dysfunctions of the venous valves and resulting reflux of blood in the superficial veins of the legs. Its clinical importance is often ignored despite being a common disease. A population-based, cross-sectional study showed 22.6% of participants had varicose veins in Clinical, Etiology, Anatomic, Pathophysiology (CEAP) grade C2-6, and venous disorders had significant association with several leg symptoms such as itching, feeling of heaviness or tightness^5^.

Lumbar spinal disease and CVD present similar clinical symptoms on legs, but there are no clear and acceptable symptomatic criteria to differentiate two conditions and their clinical relationship has not been addressed due to different clinical disciplines associated with each disease. The present study was planned to determine the incidence of CVD among patients visiting a spine center for leg pain, to distinguish it from lumbar spinal disease, and to highlight the importance of early-stage CVD as a potential cause of leg pain.

## Material and method

This study was designed as a cross-sectional study to investigate the prevalence of CVD among patients with leg pain visiting a spine center and to compare it with lumbar spinal disease. All methods were carried out in accordance with relevant guidelines and regulations and informed consent was obtained from all subjects and/or their legal guardians.

The inclusion criteria were based on patients’ symptoms and history of illness. Patients were included if they experienced leg pain combined with common clinical features of CVD, such as atypical radiculopathy mismatching dermatome level, cutaneous changes like telangiectasia, reticular veins or varicose changes, cramping, coldness, morning heaviness, nocturnal aggravation of symptoms, and chronic leg pain persisting over years.

The exclusion criteria were applied to patients presenting with definite radiculopathy symptoms and root irritation signs examined by straight leg raising test and femoral nerve stretching test, along with corresponding motor weakness. Cases with a history or diagnosis of arterial occlusion or deep vein thrombosis in the lower extremities were also excluded.

A total of 196 patients were recruited and underwent lower extremity ultrasound examinations from January to December 2021. All ultrasound examinations were performed by the first author, who had more than six months of experience in CVD ultrasound diagnosis.

The sample size of 196 patients was smaller than the calculated value for a 95% confidence level and a 5% margin of error. However, it was deemed to sufficient to provide initial insights into the study objectives.

The CEAP grade is a well-known clinical grade system for CVD that classifies it as follows: C0: no visible or palpable signs, C1: telangiectasias (veins less than < 1 mm), reticular veins (1–3 mm in diameter), C2: varicose veins (> 3 mm), C3: edema, C4: secondary skin alterations. C4a: pigmentation and eczema, C4b: lipodermatosclerosis or white atrophy, C5: healed ulcer, and C6: open ulcer^6^. All patients’ CEAP grades were recorded and classified.

The areas of symptom complaints were classified as low back, buttock, thigh, knee, calf, and foot. The types of symptoms were classified as pain, tingling-paresthesia, hypoesthesia, cramping, coldness, heat, swelling, and weakness. Each patient could have more than one symptom area and type of symptom manifestation.

The CVD diagnosis groups were classified according to the ultrasound findings, which were classified as follows: VD0 for no CVD finding, VD1 for venous reflux only in tributaries and perforators, VD2 for venous reflux in major veins such as great saphenous vein (GSV) or small saphenous vein (SSV) and varicose vein, and VD3 for venous reflux in major veins, tributaries, and perforators.

The lumbar disc herniation and the lumbar spinal stenosis were included in the lumbar spinal disease diagnosis group based on magnetic resonance imaging (MRI) findings. The lumbar disc herniation was categorized according to the degree of disc protrusion. The lumbar spinal stenosis was graded based on the presence of cerebrospinal fluid (CSF) signal as follow: Grade A for clearly visible CSF inside the dural sac, Grade B for individualized rootlets occupying the entire dural sac, Grade C for homogeneous gray signal dural sac with no visible CSF signal and unrecognizable rootlets, and Grade D for the absence of posterior epidural fat^7^. The lumbar spinal disease groups were classified by combining severities of two diseases as follows: LD0 for no definite disc protrusion or spinal canal narrowing, LD1 for mild disc bulging or stenosis grade A and B which unlikely to cause any leg symptoms, LD2 for moderate disc protrusion or stenosis grade C which potentially to cause leg pain, and LD3 for ruptured disc or stenosis grade D.

None of cases had any history or diagnosis of arterial occlusion or deep vein thrombosis in the lower extremities.

CVD-diagnosed cases (VD123) were divided into two groups for comparison analysis based on the severity of lumbar spinal disease. The first group, CVD-dominant group (VDG), had a lower degree of lumbar spinal disease and symptoms were believed to be caused by CVD. The second group, lumbar spinal disease-dominant group (LDG), had a higher degree of lumbar spinal disease, and patients’ symptoms were thought to be influenced by the lumbar spinal diseases.

Vasoactive medications were provided to the cases diagnosed with CVD, but surgical management for CVD was not performed, which could be a limitation in improving symptoms, especially in severe cases of CVD. To compensate for this limitation, the trends of symptom improvement graphs were compared between the two groups.

Symptom improvements were assessed at the first follow-up visits within a two-month period and were categorized as excellent, good, or poor based on the patients’ own descriptions of their symptoms. The visual analog scale (VAS) or numeric rating scale (NRS) was irrelevant and not used because CVD symptoms include pain as well as a variety of other symptoms. It was hypothesized that if there was significant difference in vasoactive medication effect between the VDG and LDG, it could provide indirect evidence for the existence of venous pain in patients presenting to the spine center with leg pain.

The estimation of vasoactive medication effect could be affected by several factors such as pain medication, neuropathic medication, pain block, and lumbar surgery. Pain medications (PM) were graded according to its strength as follows: PM0 for no pain medication, PM1 for NSAIDs (non-steroidal anti-inflammatory drugs), PM2 for weak opioids (combination of codeine, acetaminophen, and ibuprofen or combination of tramadol and acetaminophen), and PM3 for strong opioids. Neuropathic medications (NM) were graded as follows: NM0 for no neuropathic medication and NM1 for gabapentin or pregabalin. These factors were included in the group comparisons to estimate the effect of vasoactive medication.

The ultrasound examinations were conducted in an erect position along the pathway of the GSV, SSV and their major tributaries from the inguinal to the lower medial calf, posterior knee to posterior calf, and lateral knee to calf. The B mode ultrasound examinations were started to find the proximal junction of the saphenous vein at the inguinal area for GSV and the posterior knee for SSV. Venous refluxes were assessed using a manual compression-and-release maneuver. An assistant made an upward venous flow by manually compressing and releasing more distal leg from the test site. Upward flow and following venous flow were examined by real-time simultaneous Color and Pulsed Wave Doppler mode. A positive finding was defined as the presence of reflux flow for more than one second of reflux flow in major saphenous veins, and for at least half a second of reflux in tributaries and perforators. The examination was carried out using both a linear probe (12L, 4.5–13 MHz, GE Healthcare) and a hockey-stick probe (8L-18i, 8–18 MHz, GE Healthcare).

The frequency data were displayed without statistical comparison. Chi-square tests were used for comparing categorical variables, and a p-value of < 0.05 was considered statistically significant. The analyses were performed using the IBM SPSS Statistics software, version 25.

### Ethical approval

The study was approved by the local ethics committee (PSSH 0475–202207-HR-10) of the Pohang Strokes and Spine Hospital in July 2022.

## Results

Among 196 inclusion cases, 54 cases (27.6%) were men and 142 (72.4%) were women, with ages ranging from 25 to 84 years old (mean 61.3 ± 12.0). Symptoms on the right leg were observed in 20.4% (40 cases) and 23.0% (45 cases) were on the left leg. Bilateral symptoms were present in 56.6% (111 cases). The most common CEAP grade was C0 (46.9%, 92 cases), and sum of C0 and C1 was 78.6% (154 cases). Most patients reported symptom duration of longer than one month (83.7%, 164 cases), with 57.7% (113 cases) lasting more than one year (Table 1).

**Table 1:** Characteristics of symptom manifestation.

Characteristics of symptom	Frequency	Percent
Symptom side		
Right	40	20.4
Left	45	23.0
Both	111	56.6
Clinical CEAP grade		
C0: no visible sign	92	46.9
C1: telangiectasia, reticular vein	62	31.6
C2: varicose vein	38	19.4
C3: edema	0	0.0
C4: skin change (pigmentation)	4	2.0
Total	196	100.0
Symptom duration		
Within 1 week	4	2.0
Within 1 month	28	14.3
1–6 Months	30	15.3
6–11 Months	21	10.7
More than 1 year	113	57.7
Total	196	100.0

The ultrasound CVD diagnosis rate were 85.7% (168 cases), with negative findings in only in 14.3% (28 cases). Within diagnosis group from VD1 to 3, VD1 accounted for 41.7% (70 cases), VD2 for 19.0% (32 cases), and VD3 for 39.3% (66 cases) (Table 2).

**Table 2:** Classification of CVD diagnosis based on the ultrasound findings.

Diagnosis group	Description	Frequency	Percent (VD 0–3)	Percent (VD 1–3)
VD0	No CVD	28	14.3	
VD1	Tributaries and perforators only	70	36.7	41.7
VD2	Major vein such as GSV, SSV or varicose vein	32	16.3	19.0
VD3	Major veins, tributaries, and perforators	66	33.7	39.3
Total		196	100.0	100.0

Upon analyzing the severities of lumbar spinal diseases, it was found that 25.5% of cases did not exhibit any lumbar spinal disease (LD0: 50 cases). LD1 was present in 11.7% (23 cases), LD2 in 30.6% (60 cases), and LD3 in 32.1% (63 cases). Assuming LD2-3 could cause significant leg pain, 62.7% (123 cases) of the cases were found to be affected by lumbar spinal diseases.

Electromyography (EMG) was performed on 76 cases, accounting for 38.8% of the total cases, and radiculopathy findings were present in 50.0% (38 cases). Most of the cases with radiculopathy were LD2 and LD3, indicating that the most common accompanying disease was lumbar spinal disease (Table 3).

**Table 3:** EMG findings according to the classification of lumbar spinal disease.

LD group	Negative	Radiculopathy	Peripheral neuropathy	Total
LD0: no	2	0	0	2 (2.6%)
LD1: mild	6	1	2	9 (11.8%)
LD2: moderate	17	7	0	24 (31.6%)
LD3: severe	9	30	2	41 (53.9%)
Total	34 (44.7%)	38 (50.0%)	4 (5.3%)	76 (100.0%)

**Figure 1:**
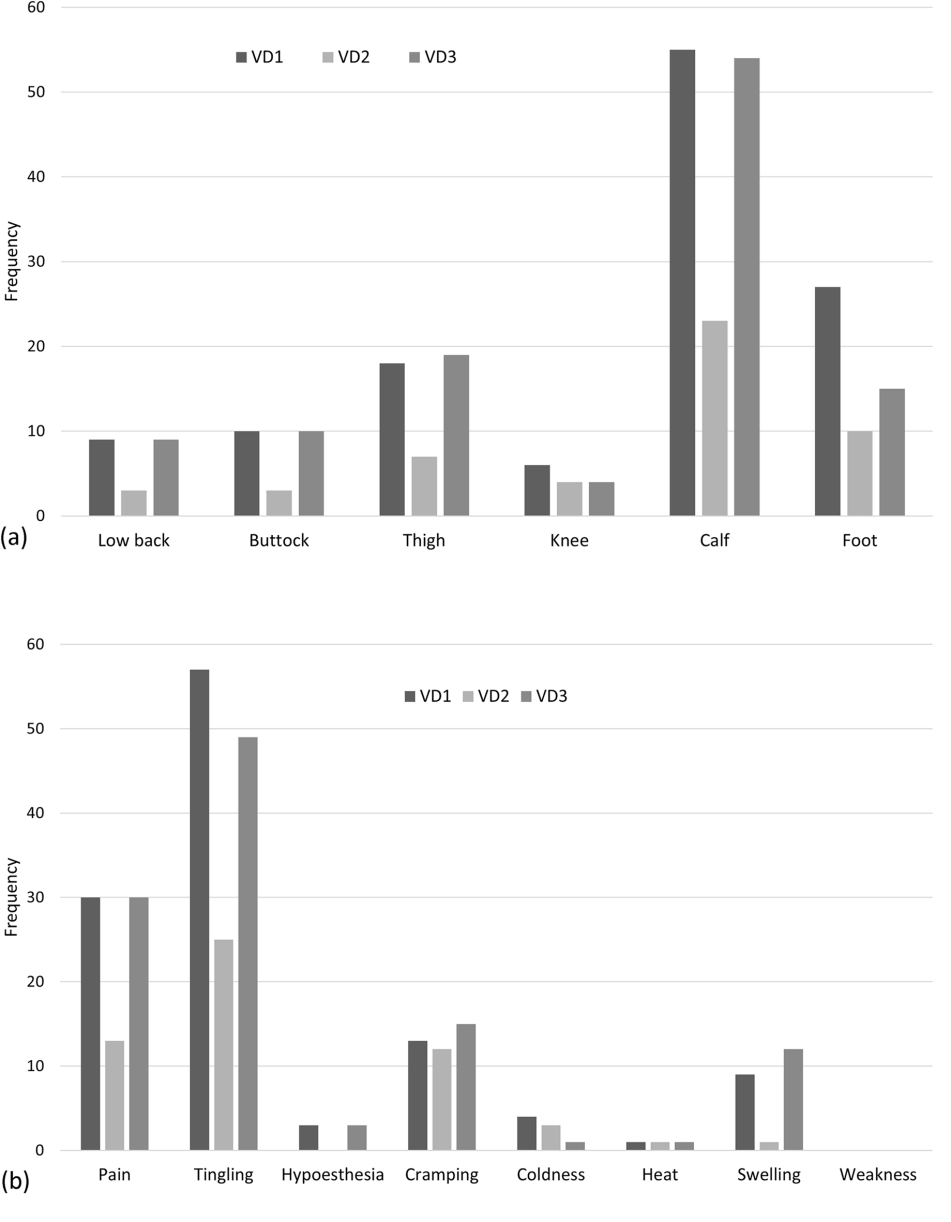
Frequency of symptom manifestations by area (**a**) and type (**b**) in cases diagnosed with CVD. Each patient could have reported more than one area and type of symptom complaint.

The frequencies of symptom manifestations were analyzed for 168 cases with a diagnosis of CVD. The most common symptom area was the calf, followed by the foot and thigh. The most common type of symptom complaint was tingling-paresthesia, followed by pain and cramping. Heat, hypoesthesia, and weakness were not common manifestations (Fig. 1).

No association was found between the distribution of CVD diagnosis groups and the severity of lumbar spinal diseases (*p* > 0.05) (Table 4). This means that the VDG and LDG comparison analyses had no significant differences in the distributions of CVD between the two groups.

**Table 4:** Comparison of CVD diagnosis group and severity of lumbar spinal disease.

Comparison	VD0	VD1	VD2	VD3	Total	*p* value
LD0	7	16	8	19	50	0.248
LD1	3	7	5	8	23	
LD2	6	17	12	25	60	
LD3	12	30	7	14	63	
Total	28	70	32	66	196	

Two cells (12.5%) have expected count less than five.

**Figure 2:**
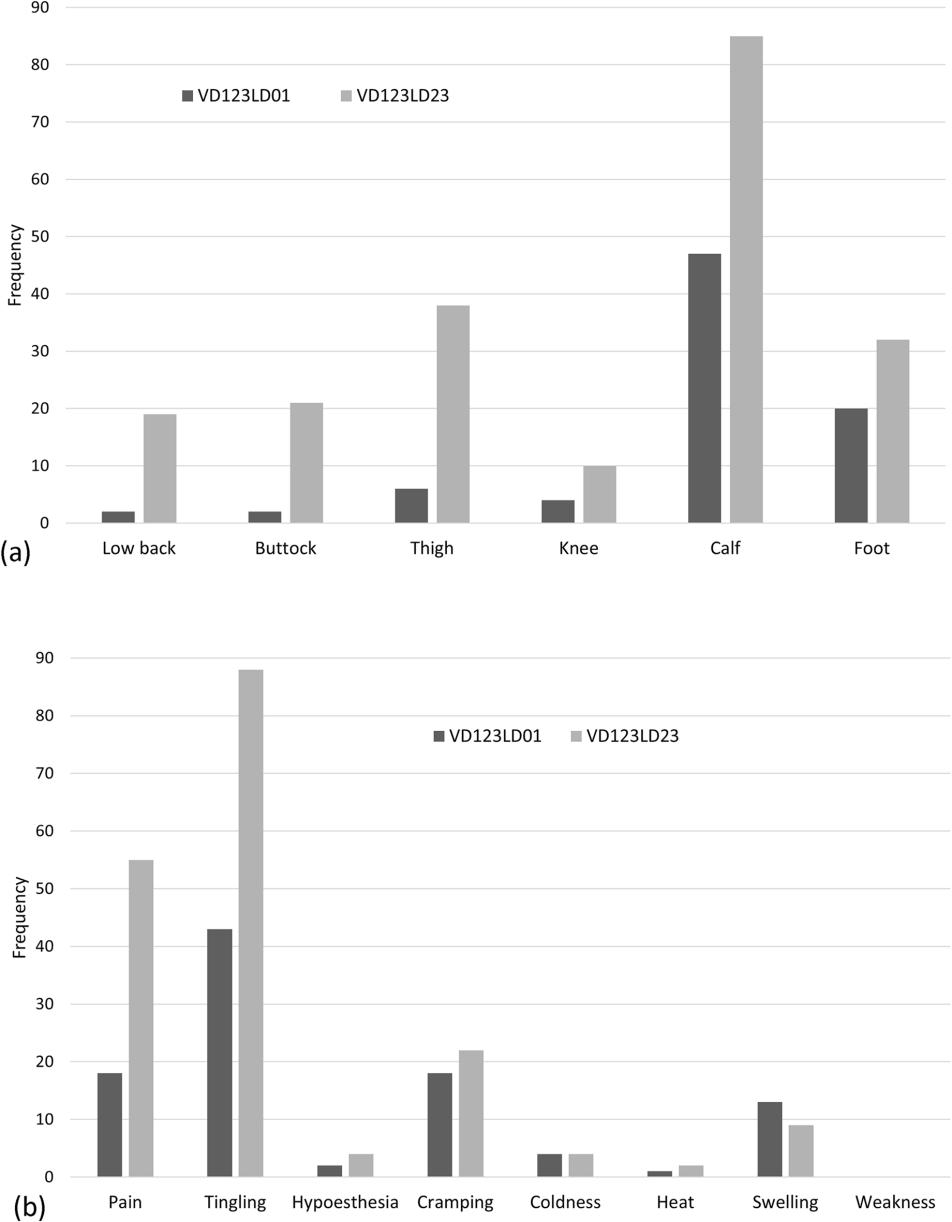
Difference in area (**a**) and type (**b**) of symptom manifestations between VDG (VD123-LD01) and LDG (VD123-LD23).

When comparing the frequencies of symptom manifestations between the VDG (VD123-LD01) and LDG (VD123-LD23), it was found that the calf was the most common symptom area for both groups. In the VDG, the second most common area was the foot, whereas in the LDG, it was the thigh. Regarding the types of symptoms, tingling-paresthesia was the most common for both groups, In the VDG, pain and cramping occurred with similar frequencies as the second most common symptoms, while in the LDG, pain was more common than cramping (Fig. 2).

From 168 CVD cases, a total of 145 cases (86.3%) were enrolled for the vasoactive medication effect evaluation except 23 cases (13.7%) of follow-up loss. Excellent symptom improvements were observed in 44.1% (64 cases), while 29.0% (42 cases) were rated as good, and 19.9% (39 cases) were rated as poor.

**Figure 3:**
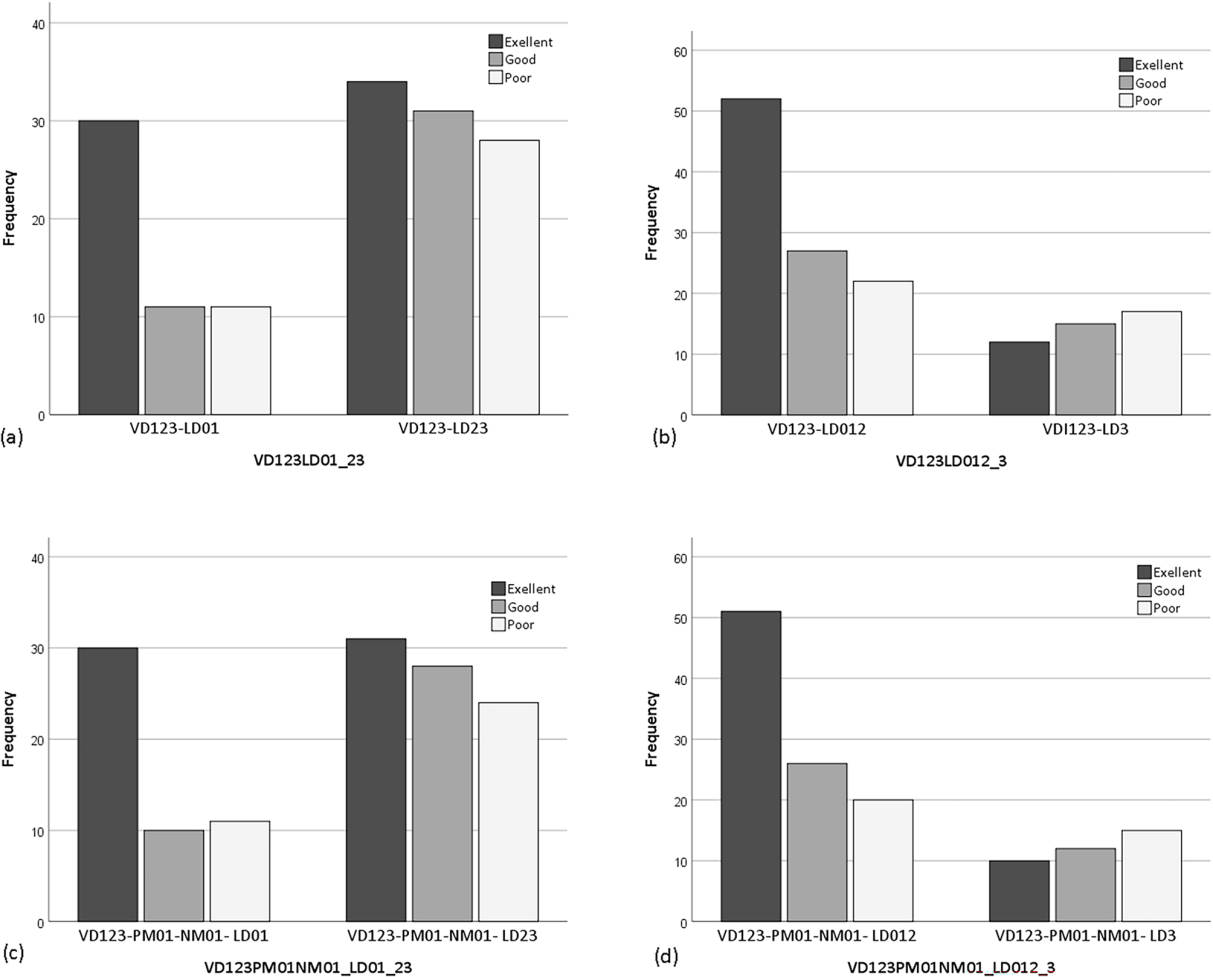
Symptom improvement comparison by four different group definitions. The first groups represent the VDG, and the second groups represent the LDG. (**a**) VD123-LD01 vs. LD23, (**b**) VD123-LD012 vs. LD3, (**c**) VD123- PM01-NM01-LD01 vs. LD23, (**d**) VD123-PM01-NM01-LD012 vs. LD3.

There was a statistically significant difference in symptom improvement between the VDG (VD123-LD01) and LDG (VD123-LD23). The rate of excellent symptom improvement rate was much higher in the VDG (57.7%, 30 cases) than in the LDG (36.6%, 34 cases). When the definition of group comparison was changed to the VDG (VD123-LD012) and LDG (VD123-LD3), the difference was similarly significant (Fig. 3a, b, Table 5a, b).

**Table 5:** Symptom improvement comparison by various group definitions.

Group		Symptom improvement				*p* value
		Excellent	Good	Poor	Total	
(a)	VD123 LD01	30 (57.7%)	11 (21.2%)	11 (21.2%)	52 (100%)	0.048*
	VD123 LD23	34 (36.6%)	31 (33.3%)	28 (30.1%)	93 (100%)	
(b)	VD123 LD012	52 (51.5%)	27 (26.7%)	22 (21.8%)	101 (100%)	0.019*
	VD123 LD3	12 (27.3%)	15 (34.1%)	17 (38.6%)	44 (100%)	
(c)	VD1 LD01	14 (66.7%)	2 (9.5%)	5 (23.8%)	21 (100%)	0.019*
	VD1 LD23	13 (31.0%)	14 (33.3%)	15 (35.7%)	42 (100%)	
(d)	VD23 LD01	16 (51.6%)	9 (29.0%)	6 (19.4%)	31 (100%)	0.640
	VD23 LD23	21 (41.2%)	17 (33.3%)	13 (25.5%)	51 (100%)	
(e)	VD123 LD01 PM0 NM0	25 (62.5%)	7 (17.5%)	8 (20.0%)	40 (100%)	0.350
	VD123 LD23 PM0 NM0	12 (50.0%)	8 (33.3%)	4 (16.7%)	24 (100%)	
(f)	VD123 LD012 PM0 NM0	35 (59.3%)	14 (23.7%)	10 (16.9%)	59 (100%)	0.442
	VD123 LD3 PM0 NM0	2 (40.0%)	1 (20.0%)	2 (40.0%)	5 (100%)	
(g)	VD123 LD01 PM01 NM0	29 (59.2%)	9 (18.4%)	11 (22.4%)	49 (100%)	0.184
	VD123 LD23 PM01 NM0	27 (43.5%)	20 (32.3%)	15 (24.2%)	62 (100%)	
(h)	VD123 LD012 PM01 NM0	48 (53.3%)	24 (26.7%)	18 (20.0%)	90 (100%)	0.200
	VD123 LD3 PM01 NM0	8 (38.1%)	5 (23.8%)	8 (38.1%)	21 (100%)	
(i)	VD123 LD01 PM01 NM01	30 (58.8%)	10 (19.6%)	11 (21.6%)	51 (100%)	0.048*
	VD123 LD23 PM01 NM01	31 (37.3%)	28 (33.7%)	24 (28.9%)	83 (100%)	
(j)	VD123 LD012 PM01 NM01	51 (52.6%)	26 (26.8%)	20 (20.6%)	97 (100%)	0.016*
	VD123 LD3 PM01 NM01	10 (27.0%)	12 (32.4%)	15 (40.5%)	37 (100%)	

The first groups represent the VDG, and the second groups represent the LDG (*p < 0.05).

When variables of PM and NM were included, significant differences were found in comparisons between the VDG and LDG (Fig. 3c, d, Table 5i, j).

When only VD1 was selected instead of VD123, it had statistically significant difference. However, the difference was not significant when VD23 was selected for the comparison (Table 5c, d).

Other comparisons for PM0-NM0 and PM01-NM0 did not show statistically significant differences, but the rates of excellent symptom improvement were still prominent (Table 5e–h).

The lumbar pain block procedures were performed in 13.8% (20 cases) of the 145 enrolled cases. This variable had a low value as a separate interfering factor for the group comparison because most pain block cases also took pain medications. The influence of pain block variable could be included in the pain medication variable.

The lumbar surgery was performed on 6.2% (9 cases) of the 145 enrolled cases within six months of vasoactive medication. It was assumed the frequency of lumbar surgery was too small to have a significant impact on the outcome of symptom improvement.

## Discussion

CVD is a common pathologic condition, but its clinical importance is not widely recognized. In a survey conducted in Belgium and Luxembourg, a total of 6009 patient were included by 406 general practitioners and the prevalence (C1-6) was found to be high at 61.3%^8^. The Vein Consult Program enrolled 91,545 subjects through 6232 general practitioners, the worldwide prevalence was found to be 83.6% (C1-6: 63.9%, C0: 19.7%), and 41.4% of these patients had early-stage CVD (C0–1)^9^. The present study focused on patients who visited a spine center due to leg pain and related symptoms. The prevalence of C1-6 was 53.1%, with higher than C2 was only 21.4%. Early-stage CVD (C0-1: 78.6%) was more dominant than other grades, indicating that a significant proportion of patients with C0-1 had leg symptoms. Patients with higher than C2 were more likely to seek venous treatment rather than visit a spine center, as venous pathology was more visible, and patients were able to recognize problems related to CVD.

The Bonn Vein Study indicated that C2-6 was associated with a feeling of heaviness, tightness, swelling and itching, C3-6 was pain while walking, and C3-4 was muscle cramp^5^. Another study showed that heavy legs, pain, and sensation of leg swelling were the most common complaints among the 3889 symptomatic patients^8^. While it is obvious that CVD is a major cause of various leg symptoms, more than half (62.7%) of the cases in this study were found to be influenced by lumbar spinal disease. Although the symptoms of two diseases were similar, characteristic differences could be found (Fig. 2). If a patient has been experiencing tingling and cramping symptoms from the calf to foot for over a year, CVD should be considered as a possible diagnosis option instead of lumbar spinal disease.

**Figure 4:**
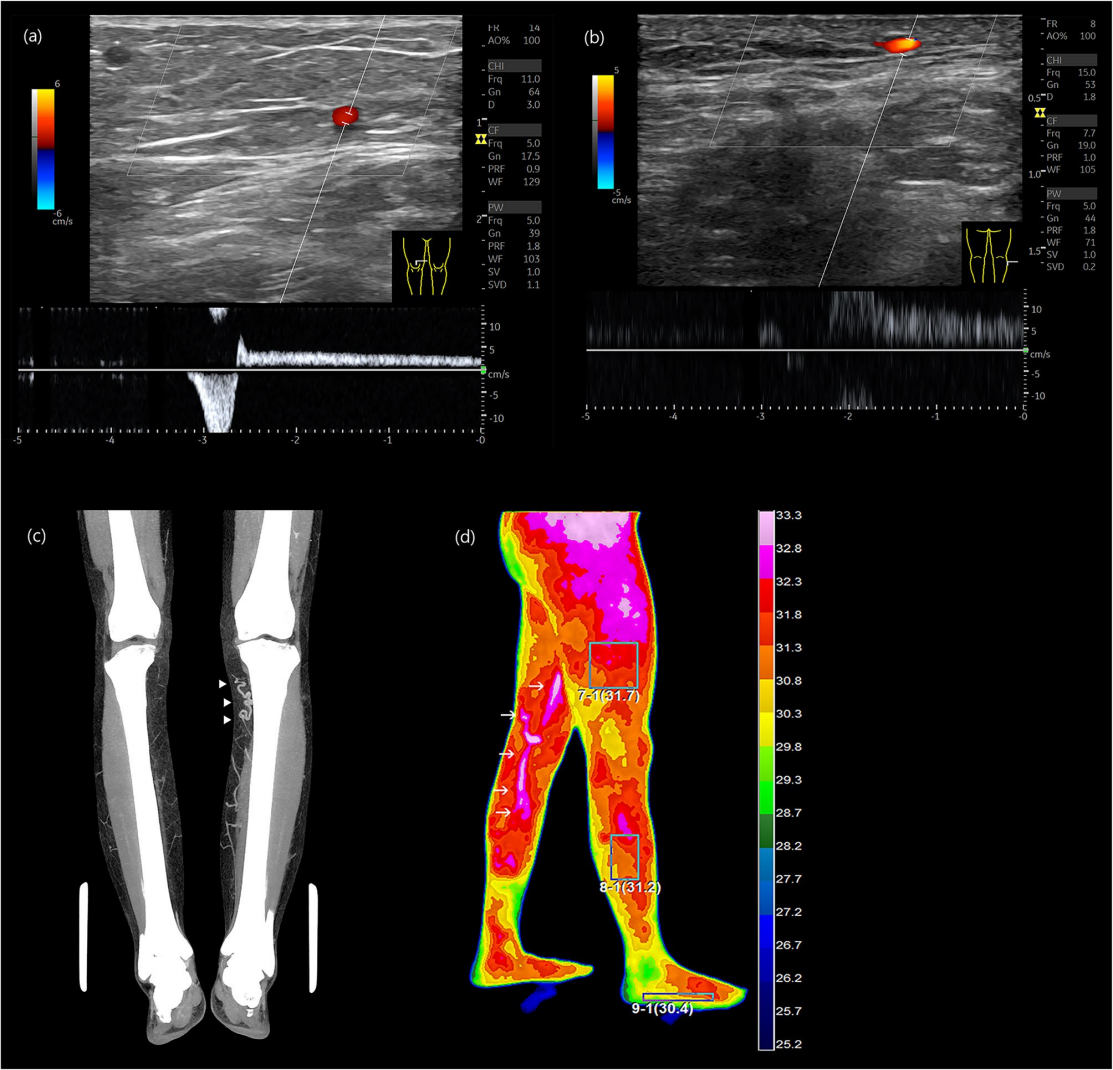
Diagnosis of CVD. (**a**) Axial ultrasound image showing GSV reflux at right lower thigh, (**b**) Axial ultrasound image displaying direct tributary reflux at right lateral calf, (**c**) Venous phase of lower extremity CT revealing a tortuous GSV at left upper calf indicating varicose change (arrowhead), (**d**) DITI showing GSV reflux at left medial thigh and calf (arrow).

The standard tool for CVD confirmation is visualizing venous reflux wave on the duplex ultrasound examination (Fig. 4a)^10, 11^. Essential procedures include visualization of the venous flow, provocative maneuvers to assess reflux, and augmentation of flow. Recent advance of ultrasound resolution and fine probes have made it possible to detect small symptomatic reflux flow as small as 1–2 mm in size (Fig. 4b).

The contrast-enhanced lower extremity computed tomography (CT) scan is useful for visualizing entire vascular pathway and tortuous varicose change of leg veins (Fig. 4c). It has superior capability than the ultrasound for visualizing complex saphenofemoral junction and superficial venous pathway variation. It is useful to find other vascular conditions such as arterial stenosis, occlusion, and deep vein pathology. Digital Infrared Thermographic imaging (DITI) is also a useful auxiliary diagnostic tool. Elevated temperature signal changes along known venous pathways or symptomatic locations are strong clues for the presence of venous reflux (Fig. 4d).

The diagnosis rate in the present study was much higher (85.7%) than in other studies, because the reflux confined to tributaries and perforators was included as a diagnosis group (VD1). Up to 41.7% of the diagnoses were due to refluxes in tributaries and perforators (VD1), while 58.3% were in major veins (VD2-3) (Table 2). As the VD1 group does not have varicose change or advanced cutaneous change, it can be included in early-stage CVD. It was already questioned that refluxes in superficial vein tributaries were largely unrecognized in symptomatic patients in 1999^12^. The comparison analysis of symptom improvement for VD1 showed significant differences, indicating that these small refluxes could be a causative factor of the leg pain (Table 5-c).

For the lumbar spinal disease, it is obvious that acute and chronic disc herniation disease can cause leg pain, tingling, and weakness. The lumbar spinal stenosis, whereas, has a more complex relationship with spinal canal narrowing and symptom correlation. The ligamentum flavum thickness was found to be correlated with the Oswestry Disability Index (ODI), and the spinal and dural sac space was associated with neurological claudication^13^. The minimum cross-sectional area of cauda equina was a strong predictor of the walking ability, leg and back pain, and quality of life^14^. While the association between the degree of stenosis and the severity of clinical symptom was questioned, stenosis Grade C and D are still considered candidates for the surgical management and are accepted as definite and symptomatic stenosis lesions^7, 15^.

Although excluded in the present study, other vascular diseases such as peripheral arterial disease and deep vein thrombosis can cause similar leg pain that worsens with walking. However, femo-gluteal fatigability or pain and impotence, which are early signs of Leriche’s syndrome, have also been attributed to co-existing lumbar spinal disease^16^. Common degenerative leg diseases such as knee or hip arthrosis can also present typical symptoms of CVD^4^.

Already progressed and clinically clear CVD patients will seek vascular diagnosis and treatment. However, early-stage CVD usually doesn’t have noticeable cutaneous changes, and patients may be unaware of the underlying the cause of their pain. These symptoms can easily be mistaken for lumbar spinal disease or considered as a part of the lumbar symptoms during the assessment of the condition. Considering that the majority of cases in the present study (78.6%) were C0-1 grade and almost half of the diagnosed cases (41.7%) were in the VD1 group, significant differences in symptom improvement between VDG and LDG provide evidence for the presence of venous pain in early-stage CVD. Simply, clinically hidden small or early-stage venous refluxes can be a cause of leg pain.

When cases taking pain and neuropathic medications were excluded from the comparison, the count of LDG was prominently lower than that of VDG (Table 5e, f). This difference can be attributed to the fact that LDG participants were already taking pain and neuropathic medications. Sequential inclusion of weak pain medications (PM01) and neuropathic medications (NM01) yielded similar counts for both the VDG and LDG, as compared to when PM and NM factors were not included. The inclusion of PM and NM factors did not lead to improved comparison results.

The present study had several limitations. It was non-randomized and observational study. A control group without CVD was not included for specific comparison. Only short-term medical treatment was included, and more invasive treatment was not. The comparison variables were categorial, further statistical analysis using continuous variables should be developed. Randomized case–control trials should be evaluated to provide more definite conclusions.

## Conclusion

CVD is not uncommon condition among patients visiting a spine center for leg pain. While the clinical presentation of CVD is similar to that of lumbar spinal disease, it has a few distinguishable clinical features. CEAP C0-1 grades and refluxes confined solely to tributaries and perforators are not asymptomatic condition, it can cause leg pain and various other symptoms. Evaluating early-stage CVD can reveal another clinically hidden cause of leg pain when assessing the lumbar spinal disease.

## Data Availability

The datasets used and/or analyzed during the current study available from the corresponding author on reasonable request.
